# Respiratory acidosis and O_**2**_ supply capacity do not affect the acute temperature tolerance of rainbow trout (*Oncorhynchus mykiss*)

**DOI:** 10.1093/conphys/coae026

**Published:** 2024-05-16

**Authors:** Daniel W Montgomery, Jennifer Finlay, Stephen D Simpson, Georg H Engelhard, Silvana N R Birchenough, Rod W Wilson

**Affiliations:** Biosciences, Stocker Road, University of Exeter, Exeter, EX4 4QD, UK; Biosciences, Stocker Road, University of Exeter, Exeter, EX4 4QD, UK; Biosciences, Stocker Road, University of Exeter, Exeter, EX4 4QD, UK; International Marine Climate Change Centre (iMC3), Centre for Environment, Fisheries & Aquaculture Science (Cefas), Pakefield Road, Lowestoft, NR33 0HT, UK; School of Environmental Sciences, University of East Anglia, Norwich, NR4 7TJ, UK; International Marine Climate Change Centre (iMC3), Centre for Environment, Fisheries & Aquaculture Science (Cefas), Pakefield Road, Lowestoft, NR33 0HT, UK; Biosciences, Stocker Road, University of Exeter, Exeter, EX4 4QD, UK

**Keywords:** CO_2_, CT_max_, hypercapnia, hyperoxia, OCLTT

## Abstract

The mechanisms that determine the temperature tolerances of fish are poorly understood, creating barriers to disentangle how additional environmental challenges—such as CO_2_-induced aquatic acidification and fluctuating oxygen availability—may exacerbate vulnerability to a warming climate and extreme heat events. Here, we explored whether two acute exposures (~0.5 hours or ~72 hours) to increased CO_2_ impact acute temperature tolerance limits in a freshwater fish, rainbow trout (*Oncorhynchus mykiss*). We separated the potential effects of acute high CO_2_ exposure on critical thermal maximum (CT_max_), caused via either respiratory acidosis (reduced internal pH) or O_2_ supply capacity (aerobic scope), by exposing rainbow trout to ~1 kPa CO_2_ (~1% or 10 000 μatm) in combination with normoxia or hyperoxia (~21 or 42 kPa O_2_, respectively). In normoxia, acute exposure to high CO_2_ caused a large acidosis in trout (blood pH decreased by 0.43 units), while a combination of hyperoxia and ~1 kPa CO_2_ increased the aerobic scope of trout by 28%. Despite large changes in blood pH and aerobic scope between treatments, we observed no impacts on the CT_max_ of trout. Our results suggest that the mechanisms that determine the maximum temperature tolerance of trout are independent of blood acid–base balance or the capacity to deliver O_2_ to tissues.

## Teaser

Experimental evidence demonstrated that the upper temperature tolerances of rainbow trout were unaffected by acute exposure to elevated CO_2_ when combined with normoxia or hyperoxia, indicating that the mechanisms underlying temperature tolerance were not sensitive to either blood acid–base balance or O_2_ delivery.

## Introduction

Fluctuations in temperature within aquatic ecosystems have physiological impacts on ectotherms, including fish. The steady warming of freshwater (O’Reilly *et al.,* 2015) and marine systems (Lyman *et al.,* 2010; Johnson and Lyman, 2020) due to climate change, as well as the increase in frequency and severity of extreme temperature events (Oliver *et al.,* 2018; Perkins-Kirkpatrick and Lewis, 2020; Woolway *et al.,* 2021), exacerbates the thermal challenges individual fish may face in the future. Knowledge of a species’ temperature tolerance limits can be used to inform management and conservation measures (Macdonald *et al.,* 2012; Breau and Caissie, 2013; Pimentel *et al.,* 2015), predict shifts in population distributions and ranges (Sunday *et al.,* 2012; Dahlke *et al.,* 2020) and is increasingly needed as climate change advances. However, the mechanisms that determine the temperature tolerance limits of fish have yet to be fully determined, which presents problems for predicting responses to climate change. Additionally, there are other environmental factors changing simultaneously with warming, such as elevated CO_2_ or hypoxia, which often co-occur with periods of high temperatures (Brauko *et al.,* 2020; Tassone *et al.,* 2022), and it is unknown if these other physiological challenges can also affect temperature tolerances.

Whether elevated CO_2_ and hypoxia can influence thermal tolerance limits depends on the physiological mechanisms underlying critical temperature tolerance limits. One prominent hypothesis, ‘oxygen- and capacity-limited thermal tolerance’ (OCLTT) (Pörtner and Knust, 2007), predicts that the upper temperature tolerance limits of fish occur once the cardiorespiratory system is unable to increase O_2_ supply to meet rising tissue O_2_ demands. This mismatch is expected to cause tissue hypoxaemia and dependence on unsustainable anaerobic metabolism for survival. The OCLTT hypothesis would therefore predict that changes in environmental O_2_ and CO_2_ levels would affect the thermal tolerance limits of fish via direct impacts on the O_2_ supply capacity of ectotherms (Pörtner *et al.,* 2017). However, studies to date have not universally supported the OCLTT hypothesis, with impacts of environmental hypoxia and hyperoxia (Devor *et al.,* 2016; Ern *et al.,* 2016; Giomi *et al.,* 2019; Islam *et al.,* 2020; Leeuwis *et al.,* 2021; Andreassen *et al.,* 2022; McArley *et al.,* 2022a; Nati *et al.,* 2023), chronic high CO_2_, and direct manipulations of physiological O_2_ supply capacity (Brijs *et al.,* 2015) showing species-specific effects on temperature tolerance. A recent review has highlighted that complex inter-related physiological systems could determine the upper thermal tolerance limits of fish (Ern *et al.,* 2023) and suggests several alternative underlying mechanisms that may determine the critical thermal maximum (CT_max_). These alternative mechanisms, such as changes in protein function and membrane fluidity, may not be affected by oxygen availability but could be impacted by other environmental factors, including exposure to elevated environmental CO_2_ during periods of high temperature.

While chronic exposure (days to weeks) to elevated aquatic CO_2_ levels has not previously been shown to impact the thermal tolerance of fish (Clark *et al.,* 2017; Ern *et al.,* 2017), to our knowledge, no studies to date have examined the acute impacts of high CO_2_ (minutes to hours) on thermal tolerance limits. However, in marine and freshwater environments, fish may well experience abrupt elevations in environmental CO_2_ (often many magnitudes greater than levels used during chronic CO_2_ experiments). Exposure to acutely elevated CO_2_ can occur for a variety of reasons, for example, by moving into areas where enhanced metabolic CO_2_ production has occurred (Melzner *et al.,* 2013; Baumann *et al.,* 2015), as a result of upwelling events (Feely *et al.,* 2008), increased nutrient inputs, and eutrophication (Sunda and Cai, 2012; Morales-Williams *et al.,* 2021). Alternatively, direct inputs of CO_2_ into the environment can occur from natural sources (Hall-Spencer *et al.,* 2008; Kerrison *et al.,* 2011) or due to management interventions (Suski, 2020; Zolper *et al.,* 2022). Crucially, the absence of effects of chronic CO_2_ exposure on the thermal tolerance of fish does not preclude impacts of acute CO_2_ exposure on thermal tolerance as fish acutely exposed to elevated CO_2_ suffer respiratory acidosis (i.e. decreased pH of extracellular and intracellular fluids), which causes major physiological impacts and is subsequently regulated during chronic exposure.

Respiratory acidosis typically reduces the O_2_ supply capacity of fish by decreasing the O_2_-binding affinity of pH-sensitive haemoglobins (Hannan and Rummer, 2018; Brauner *et al.,* 2019), but it can also impact the performance of other physiological processes. Small reductions in internal pH caused by respiratory acidosis can have significant effects on tissue function, including cardiac and brain tissue (Gesser and Poupa, 1983; Farrell and Milligan, 1986; Siesjö *et al.,* 1993) and enzyme activity (Yang and Honig, 1993). Since the OCLTT hypothesis has not yet been shown universally to determine the upper temperature tolerance limits of fish, it is possible that alternative mechanisms could be involved, including disruption of neural function (Ern *et al.,* 2015; Jutfelt *et al.,* 2019; Andreassen *et al.,* 2022), impairment of cell membranes (Bowler, 2018), enzyme denaturation (Somero and Dahlhoff, 2008), ion imbalances (particularly of potassium) (O’Sullivan *et al.,* 2017; Zimmer *et al.,* 2024) and failure of critical organs (e.g. heart or brain) (Sidhu *et al.,* 2014; Jørgensen *et al.,* 2017; Andreassen *et al.,* 2022; Schwieterman *et al.,* 2023). Indeed, all of the above processes could be involved in determining the acute temperature tolerance of fish (Ern *et al.,* 2023), demonstrating the complexity of predicting how environmental changes may impact thermal limits. Determining whether acutely increased CO_2_ impacts upon the CT_max_ of fish may provide further information on which mechanisms underlie thermal tolerance by highlighting whether they are sensitive to internal acid–base disturbances.

In this study, we sought to determine whether acute exposure to elevated environmental CO_2_ influences the temperature tolerance of fish, using rainbow trout (*Oncorhynchus mykiss*) as a model species. We predicted that acute exposure to elevated CO_2_ would reduce the temperature tolerance of trout via one of two potential mechanisms: either via the impact of respiratory acidosis on haemoglobin (and therefore O_2_ supply capacity) or due to the impact of respiratory acidosis on physiological functions in other tissues. Our objectives for this study were to isolate the potential effects of acute high CO_2_ exposure on temperature tolerance via either respiratory acidosis or O_2_ supply capacity by manipulating environmental CO_2_ and O_2_. Specifically, by using elevated environmental O_2_ (hyperoxia) exposures to restore O_2_ supply capacity in trout which were acutely exposed to high CO_2_ without affecting respiratory acidosis. Finally, we investigated whether the change in blood chemistry due to acid–base regulation after a 72-hour exposure to elevated CO_2_ affected temperature tolerance.

## Materials and Methods

### Animal husbandry and acclimation

Immature, mixed-sex, diploid rainbow trout (~1 year old) were obtained from a commercial supplier, where they were spawned and reared in freshwater (Houghton Springs Hatchery, Dorset, UK) in three batches between January and September 2020 (Supplementary Table S1). Once transferred to Exeter, trout were held in four ~500-l holding tanks (stocking density ~10 kg m^−3^) supplied by a freshwater recirculating aquaculture system (RAS) (pH = 7.69 ± 0.07, temperature = 15.0 ± 0.3°C, salinity = 0.13 ± 0.04, mean ± SD); for general details of freshwater inorganic chemistry, see Cooper and Wilson (2008). Trout were fed three times per week at a ration of ~ 1% body mass with a commercial pellet (Horizon 40, Skretting) and held in the RAS for at least 4 weeks to acclimate to laboratory conditions and ensure that normal feeding behaviour resumed before any experimental work. All experimental procedures were carried out under the UK Home Office licence (P88687E07) and were approved by the University of Exeter’s Animal Welfare and Ethical Review Board.

### Experimental treatments

**Figure 1:**
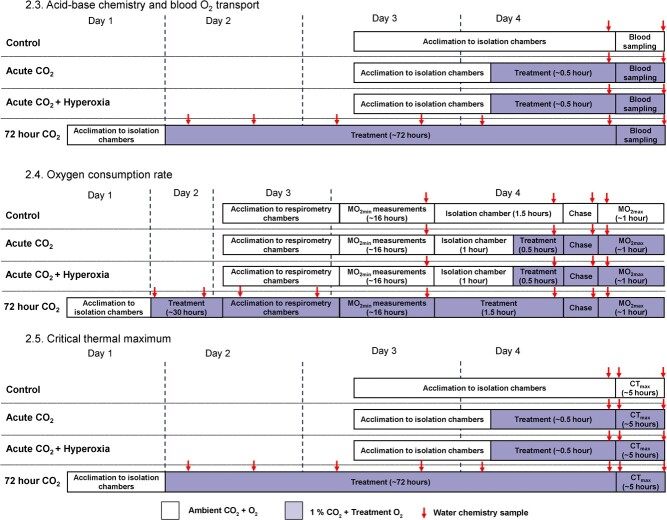
Schematic of experimental timelines for control, acute high CO_2_, acute high CO_2_ + hyperoxia and 72-hour high CO_2_ treatments within each of the three series of measurements. Clear boxes indicate times when trout were exposed to ambient CO_2_ and O_2_ conditions (i.e. control treatment), while shaded boxes indicate times when trout were exposed to 1 kPa CO_2_ and the O_2_ level relevant to the specific treatment. Red arrows indicate times when water chemistry was measured during each treatment or measurement.

Prior to experimental treatments, food was withheld for a minimum of five days to ensure physiological responses were not affected by processes associated with feeding or digestion (Chabot *et al.,* 2016). All trout from within a tank were then used for experimental trials that started within three days of the minimum starvation period. Experimental trials were conducted in a semi-recirculating experimental system that included four ~20-l isolation tanks and two ~ 300-l water baths (each containing two intermittent flow respirometers). Both the isolation tanks and water baths were fed by an ~150-l experimental sump at a rate of ~4 l min^−1^ with overflowing water returning to the sump tank. The sump tank was fed from the stock RAS at a rate of ~10 l min^−1^ with overflowing water returning to the RAS. The experimental system was maintained at 15°C using a heater/chiller unit (Grant TX150 R2, Grant Instruments, Cambridge, UK). After starvation, four trout at a time were moved to the experimental system for trials (Fig. 1).

The four trout were then exposed to one of four treatments designed to isolate the effects of high CO_2_ exposure via either respiratory acidosis or oxygen supply capacity (Table 1). All four treatments were tested within each of the three batches of trout used for experiments (i.e. treatments were not confounded by batch). For control treatment isolation tanks and respirometer water baths were gassed with ambient air. For high CO_2_ treatments the experimental sump was dosed with CO_2_ by an Aqua Medic pH computer (to maintain a set pH ± 0.02 units). For acute high CO_2_ in normoxia and ~72-hour high CO_2_ treatments isolation tanks and respirometer water baths were gassed with a mix of 1% CO_2_, 21% O_2_ and 78% N_2_ (G400 Gas mixing system; Qubit Biology Inc.). For acute high CO_2_ treatments in hyperoxia, the isolation boxes, respirometer water baths and the experimental sump were gassed with a mix of 1% CO_2_, ~ 50% O_2_ and ~49% N_2_ to produce an environmental pO_2_ of ~ 42 kPa O_2_.

**Table 1:** Proposed treatment conditions to isolate the effects of high CO_2_ exposure via either O_2_ supply capacity or respiratory acidosis

Treatment	Duration (hours)	pCO_2_ (kPa)	pO_2_ (kPa)	O_2_ supply capacity	Respiratory acidosis	Predicted effect on CT_max_
Control	n/a	0.1	21	No change	χ	N/A
Acute high CO_2_ + normoxia	0.5	1	21	↓	✓	↓
Acute high CO_2_ + hyperoxia	0.5	1	42	No change	✓	= if only O_2_ supply capacity affects CT_max_ ↓ if only respiratory acidosis affects CT_max_
72-hour high CO_2_	72	1	21	No change	χ	=

### Blood acid–base chemistry and O_2_ transport

Four trout at a time were moved from stock tanks and individually housed in isolation tanks in the experimental system. After acclimation to isolation tanks for 24 hours in ambient conditions, trout were then exposed to either control, acute high CO_2_, acute high CO_2_ and hyperoxia or 72-hour high CO_2_ treatments (Fig. 1). After treatment exposure, trout used for blood sampling (control, *n* = 8; acute CO_2_, *n* = 9; acute CO_2_ + hyperoxia, *n* = 8; 72 hour CO_2_, *n* = 8) were anaesthetized *in situ* using a dose of 70 mg L^−1^ of benzocaine. Blood samples were then obtained while anaesthesia was maintained, using methods validated by Davison *et al.* (2023), with gills irrigated by water with the same CO_2_ and O_2_ content as the respective treatment. Trout were then euthanized with a dose of 250 mg∙L^−1^ of benzocaine. Immediately after sampling we measured extracellular pH (pH_e_), haematocrit (Hct), haemoglobin (Hb) content and plasma TCO_2_ before calculating plasma pCO_2_ and HCO_3_^−^ following details set out in Montgomery *et al.* (2019, 2022). Intracellular pH (pH_i_) of red blood cells (RBC) was measured using the freeze-and-thaw method (Baker *et al.,* 2009). All measurements or storage of blood occurred within 10 minutes of blood sampling. Finally, we measured Hb–O_2_ affinity and Hill’s number using a Blood Oxygen Binding System (BOBS, Loligo systems) using a gas mix with the same pCO_2_ level as calculated in plasma for each individual trout.

Measurements of water pH (NBS scale), temperature, pO_2_ and salinity, as well as water samples to measure total CO_2_ (TCO_2_)/Dissolved Inorganic Carbon (DIC), were taken from the isolation tank of each trout during treatments and from gill irrigation tables during the time of blood sampling (Fig. 1). DIC analysis was conducted using a custom-built system described in detail by Lewis *et al.* (2013) and pCO_2_ and total alkalinity calculated using CO2SYS (Pierrot *et al.,* 2006) as described in Montgomery *et al.* (2022). Water chemistry details for each treatment are given in Supplementary Table S2.

### Oxygen consumption rate

Oxygen consumption rates (ṀO_2_, mg O_2_ kg^−1^∙h^−1^) were measured using temperature compensated fibre-optic O_2_ sensors (Robust O_2_ probe & Firesting O_2_ meter, Pyro Science, GmbH, Germany) in an intermittent-flow respirometer system. For control (*n* = 12), acute high CO_2_ + normoxia (*n* = 11) and acute high CO_2_ + hyperoxia treatments (*n* = 10), trout were acclimated to the respirometer chambers for 24 hours before measurements of ṀO_2_ were made for ~ 16 hours and minimum ṀO_2_ (ṀO_2min_) estimated as the mean of the 10 lowest ṀO_2_ measurements. Water chemistry conditions in the respirometers were maintained at ambient CO_2_ and O_2_ levels during measurements for these treatments. For the 72 hour high CO_2_ treatment (*n* = 11), trout were moved to respirometers after ~ 30 hours exposure to increased CO_2_ in individual isolation boxes and acclimated to the respirometer chambers, with water chemistry maintained at ~ 1% CO_2_, for a further 24 hours before measurements of ṀO_2_ began. Measurements of ṀO_2_ were then made for ~ 16 hours and minimum ṀO_2_ (ṀO_2min_) was estimated as the mean of the 10 lowest measurements. All trout were then moved to individual isolation chambers for 1.5 hours. For trout from control treatments isolation boxes had ambient levels of CO_2_ and O_2_ for the entire 1.5 hours. For trout in acute high CO_2_ + normoxia treatments and acute high CO_2_ + hyperoxia treatments individual isolation boxes had ambient levels of CO_2_ and O_2_ for the 1^st^ hour after transfer before trout were exposed to ~ 1% CO_2_ with either ~ 21 kPa or ~42 kPa O_2_ for ~ 30 minutes. For trout from 72 hour high CO_2_ treatments isolation boxes had ~ 1% CO_2_ and ambient O_2_ for 1.5 hour. Finally, trout were transferred to a circular chase tank (60 cm diameter with ~ 15 cm water depth) with the appropriate CO_2_ and O_2_ level for each respective treatment and chased to exhaustion (cessation of burst swimming and lack of response to a light pinch of the caudal fin) before immediately being placed in respirometer chambers (which now had CO_2_ and O_2_ levels appropriate for each respective treatment) where ṀO_2_ was measured for one hour (average time from end of chase to beginning of measurements was ~ 2 minutes). Maximum O_2_ consumption (ṀO_2max_) of trout was then taken as the highest single ṀO_2_ measurement, which for all trout occurred during the first measurement period after they were placed in respirometers. As a result, ṀO_2max_ measurements for trout acutely exposed to high CO_2_ occurred at ~ 35 to 40 minutes after initial high CO_2_ exposure (i.e. at a comparable time point as measurements of acid–base chemistry and blood O_2_ transport capacity). The time course of treatment exposures and ṀO_2_ measurements is given in Fig. 1. Background respiration was measured in each empty respirometer chamber for a minimum of 1 hour at the beginning of ṀO_2min_ measurements and end of ṀO_2max_ measurements and subtracted from the measured ṀO_2_ values by assuming a linear change in background respiration over time.

Maximum and minimum ṀO_2_ measurements were then mass corrected by scaling background corrected ṀO_2_ to an average individual mass of 200 g (corresponding closely with the mean mass (200.1 ± 73.4 g, ± SD) of all trout used for ṀO_2_ measurements) using the following equation:

$$ \dot{\mathrm{M}}{\mathrm{O}}_2\ \left(200\ \mathrm{g}\right)=\dot{\mathrm{M}}{\mathrm{O}}_2\times{\left(\frac{M}{200}\right)}^{\left(1-A\right)} $$

where ṀO_2_ (200 g) is the predicted oxygen consumption of an individual scaled to a body mass of 200 g, ṀO_2_ is the measured oxygen consumption of a trout with mass of *M*, and *A* is the mass exponent describing the relationship between metabolic rate and body mass. We calculated *A* separately for ṀO_2min_ and ṀO_2max_ as the slope of the linear regression fitted through the relationship between log_10_(ṀO_2min_) or log_10_(ṀO_2max_) and log_10_(*M*) for all trout used in the study (Supplementary Fig. S1). Absolute aerobic scope (AAS) for each trout was then calculated as the difference between scaled estimates of ṀO_2min_ and ṀO_2max_. Full details of respirometry setup and data analysis can be found in Supplementary Table S3.

Water chemistry (temperature, pH, pO_2_, salinity) of the respirometry system, isolation chambers and chase chamber were measured and samples of water collected during treatments, ṀO_2min_ measurements, exhaustive exercise and ṀO_2max_ measurements (Fig. 1) to calculate carbonate chemistry conditions as detailed in section 2.3. Water chemistry details for each treatment are given in Supplementary Table S4.

### Critical thermal maximum

Two trout at a time were moved from stock tanks and individually housed in isolation tanks in the experimental system. After acclimation to isolation tanks for 24 hours in ambient conditions, trout were then exposed to either control, acute high CO_2_, acute high CO_2_ and hyperoxia, or 72 hour high CO_2_ treatments (Fig. 1). CT_max_ trials of trout (*n* = 12) from each treatment group were then conducted by switching water flow (~3 l min^−1^, 90% replacement per 15 min) to a separate heater/chiller unit (Grant TX150 R2; Grant Instruments, Cambridge, UK) with overflowing water returning to the heater/chiller unit to create a separate closed system. Temperature was then continuously increased by the heater/chiller at a rate of ~ 0.05°C minute^−1^ (3°C hour^−1^) using an automated software until CT_max_ was reached (determined as the point at which trout showed loss of equilibrium for ~ 5 seconds). We calculated the heating rate used in the CT_max_ trials in order to maintain core body temperature (T_b_) of trout at the same temperature as the surrounding water using the equation from Killen (2014):

$$ {T}_b={T}_a+\left({T}_i-{T}_a\right)\ast{e}^{- kt} $$

where T_a_ is the ambient water temperature, T_i_ is the initial water temperature, t is the time between temperature readings (mins) and *k* is the rate of change of core temperature. As we did not have a value of *k* for rainbow trout we approximated it as 0.352 for a 250 g trout using the relationship between *k* and mass (*M*) from another species (*Tilapia mossambica*) measured across fish of a similar size range to the trout used in this study (Stevens and Fry, 1970, 1974).

Water chemistry conditions of isolation boxes (temperature, pH, pO_2_, salinity) were measured and samples of water collected during treatments for carbonate chemistry analysis as in section 2.3. Throughout CT_max_ trials treatment CO_2_ and O_2_ levels were maintained by gassing the sump chamber in the heater/chiller unit with the appropriate gas mix for that treatment. The temperature and pO_2_ of each isolation chamber was monitored throughout each trial (Firesting O_2_ meter; Pyro Science, Gmbh, Germany) and measurements of pH and salinity, as well as a water sample to calculate water carbonate chemistry, were taken at the beginning and end of trials (Fig. 1). Water chemistry details for each treatment are given in Supplementary Table S5. Due to COVID-19 restrictions in place at the time of experiments only one researcher was permitted within the laboratory facility to carry out experiments and so the observer was not blinded to treatment ID during CT_max_ trials.

### Statistical analysis

All statistical analysis was conducted in R v3.6.3 (R Core Team, 2020). Changes in blood chemistry (pH_e_, pH_i_, pCO_2_, HCO_3_^−^) and O_2_ transport (Hct, Hb, Hb–O_2_ binding affinity (P_50_), Hill’s coefficient of binding cooperativity) across treatments were analysed using one-way ANOVA and assumptions of normality and equal variance of model residuals were checked by visual inspection of residual plots. If one-way ANOVA indicated significant differences between treatments, post-hoc tests were then conducted using Tukey’s Honest Significant Differences (Tukey’s HSD). Measurements that did not meet statistical assumptions of one-way ANOVA were analysed using a Kruskal–Wallis test with post hoc tests using Dunn’s test (Ogle *et al.,* 2020).

The effects of treatment on ṀO_2min_, ṀO_2max_ and AAS were analysed using general linear mixed models (GLMM) including treatment as a categorical explanatory variable and batch number as a random intercept term whereas CT_max_ was analysed using a general linear model (GLM) with treatment as a categorical explanatory variable (as all CT_max_ trials were performed in trout from the same batch). Residual diagnostic plots of each model were assessed using package ‘DHARMa’ to confirm validity of model fit (Hartig, 2020). The effect of treatment on each oxygen consumption measure was determined by chi-squared tests that compared the GLMMs which included treatment as an explanatory variable with a simplified GLMM without this variable while the effect of treatment on CT_max_ was determined in the same manner using *F* tests to compare GLMs. If treatment had a significant effect on a ṀO_2_ measurement or CT_max_, post hoc tests were performed using pairwise comparisons of least-squares means and 95% confidence intervals (95% CI) generated from package ‘emmeans’ (Lenth, 2020). Model summaries of GLM’s and GLMM’s can be found in Supplementary Table S6. All results are given as means ± standard error (S.E.) unless otherwise specified.

## Results

### Blood acid–base chemistry

**Figure 2:**
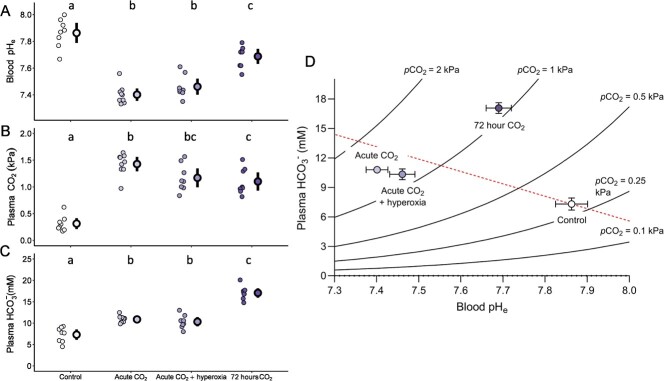
Changes in blood acid–base chemistry of trout in response to combined changes in environmental CO_2_ and O_2_. (**A**) blood pH_e_, (**B**) plasma pCO_2_, and (**C**) plasma HCO_3_^−^ of rainbow trout compared between control trout (~0.1 kPa CO_2_, ~ 21 kPa O_2_, *n* = 8), trout exposed to ~ 1 kPa CO_2_ acutely (~30 minutes) in normoxia (~21 kPa O_2_, *n* = 9) and hyperoxia (~42 kPa O_2_, *n* = 8), and for ~ 72 hours in normoxia (~21 kPa O_2_, *n* = 8). Individual circles represent raw data points for each treatment with the mean and 95% confidence interval of mean indicated by the bold outlined data point and vertical black line. Different letters above the mean of each treatment represent significant differences in post-hoc tests (*P* < 0.05). (**D**) Combined changes of all three acid–base parameters are expressed as a pH/HCO_3_^−^/pCO_2_ diagram (dashed line indicates estimated non-bicarbonate blood buffer line based on equations from Wood *et al.* (1982)) with values representing mean ± SE.

Exposure to ~ 1% CO_2_ caused significant changes to blood pH_e_ (*F* = 49.88, df = 3, *P* < 0.001), plasma pCO_2_ (*F* = 41.12, df = 3, *P* < 0.001) and plasma HCO_3_^−^ (*F* = 64.39, df = 3, *P* < 0.001). Trout exposed acutely to high CO_2_, in both normoxia and hyperoxia, experienced a large respiratory acidosis, indicated by blood pH_e_ decreasing by 0.46 (95% CI = 0.36–0.53, *P* < 0.001; Fig. 2A) and 0.40 units (95% CI = 0.30–0.48, *P* < 0.001; Fig. 2A) respectively in comparison to control trout. Reduced blood pH_e_ was a result of increased plasma pCO_2_ of trout (Fig. 2B) by 1.11 kPa in normoxia + high CO_2_ (95% CI = 0.91–1.24, *P* < 0.001) and 0.86 kPa in hyperoxia + high CO_2_ (95% CI = 0.67–1.06, *P* < 0.001). Seventy-two hour exposure to elevated CO_2_ resulted in similarly elevated plasma pCO_2_ (difference of means = 0.79 kPa, 95% CI = 0.62–0.98, *P* < 0.001). However, blood pH_e_ (Fig. 2A) had made a substantial recovery towards control levels due to increased plasma HCO_3_^−^ levels compared to trout exposed acutely to elevated CO_2_ in normoxia and hyperoxia (increased by 6.2 mM (95% CI = 5.2–7.4, *P* < 0.001) and 6.7 mM (95% CI = 5.4–8.3, *P* < 0.001) respectively (Fig. 2C). Trout exposed to high CO_2_ for 72 hours had plasma HCO_3_^−^ that was ~ 10 mM higher (95% CI = 7.79–11.72, *P* < 0.001) than control trout but had blood pH_e_ levels 0.18 (95% CI = 0.09–0.26, *P* = 0.002) lower than control trout (Fig. 2A), indicating incomplete compensation of respiratory acidosis after 72 hours (Fig. 2D).

**Figure 3:**
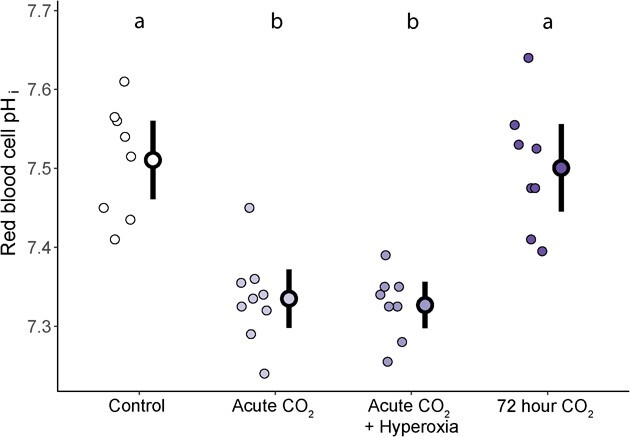
Red blood cell intracellular pH (pH_i_) of trout exposed to either control (~0.1 kPa CO_2_, ~ 21 kPa O_2_, *n* = 8), acute (~30 minutes, ~ 1 kPa CO_2_, ~ 21 kPa O_2_, *n* = 9), acute + hyperoxia (~30 minutes, ~ 1 kPa CO_2_, ~ 42 kPa O_2_, *n* = 8), or ~72 hours high CO_2_ (~1 kPa CO_2_, ~ 21 kPa O_2_, *n* = 8) treatments. Individual circles represent raw data points for each treatment with the mean and 95% confidence interval of mean indicated by the bold outlined data point and vertical black line. Different letters above the mean of each treatment represent significant differences in post-hoc tests (*P* < 0.05).

The extracellular acidosis caused by acute exposure to high CO_2_ also reduced RBC pH_i_ (*F* = 20.53, df = 3, *P* < 0.001; Fig. 3). Red blood cell pH_i_ was 0.18 lower than control conditions in trout acutely exposed to ~ 1% CO_2_ in normoxia (95% CI = 0.12–0.23, *P* < 0.001) and hyperoxia (95% CI = 0.13–0.24, *P* < 0.001). In contrast to blood pH_e_, RBC pH_i_ of trout exposed to ~ 1% CO_2_ for 72 hours was indistinguishable from control trout (difference of means = −0.01, 95% CI = −0.08—0.06, *P* = 0.757), indicating complete RBC pH_i_ regulation.

### Blood O_2_ transport

**Figure 4:**
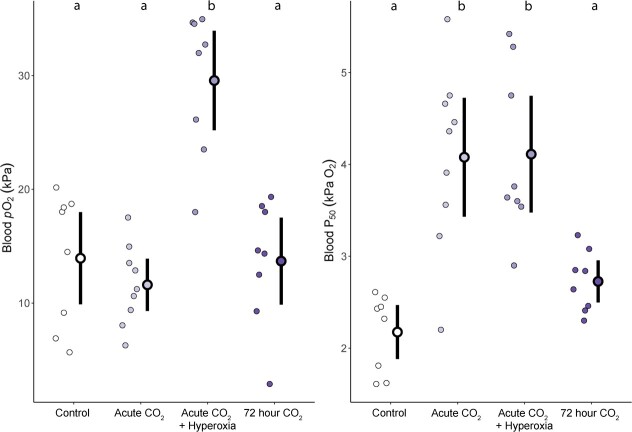
(**A**) Plasma pO_2_ and (**B**) Hb–O_2_ binding affinity (P_50_) of trout exposed to control (~0.1 kPa CO_2_, ~ 21 kPa O_2_, *n* = 8), acute (~30 minutes, ~ 1 kPa CO_2_, ~ 21 kPa O_2_, *n* = 9), acute + hyperoxia (~30 minutes, ~ 1 kPa CO_2_, ~ 42 kPa O_2_, *n* = 8), or ~72 hours high CO_2_ (~1 kPa CO_2_, ~ 21 kPa O_2_, *n* = 8) treatments. Individual circles represent raw data points for each treatment with the mean and 95% confidence interval of mean indicated by the bold outlined data point and vertical black line. Different letters above mean of each treatment represent significant differences in post hoc tests (*P* < 0.05).

There were no differences in Hct levels (*F* = 1.10, *P* = 0.367) or Hb concentration (*F* = 0.91, *P* = 0.447) of trout between treatments, with an overall mean of 35.3% and 5.51 mM, respectively. Blood pO_2_ levels did differ between treatments (χ^2^ = 16.39, *P* = 0.001; Fig. 4A) with higher pO_2_ in trout exposed to acute high CO_2_ with hyperoxia (29.6 ± 2.2 kPa) when compared to trout exposed to control CO_2_ with normoxia (difference of means = 15.6 kPa O_2_, 95% CI = 9.9–20.8, *P* = 0.010), acute high CO_2_ with normoxia (difference of means = 18.0 kPa, 95% CI = 12.8–22.0, *P* < 0.001) and ~72 hours high CO_2_ with normoxia (difference of means = 15.9 kPa, 95% CI = 10.4–21.3, *P* = 0.011).

Acute high CO_2_ exposure caused changes in P_50_ (*F* = 14.41, df = 3, *P* < 0.001), with trout acutely exposed to high CO_2_ in both normoxia and hyperoxia showing higher P_50_ values than control trout, difference of means = 1.90 (95% CI = 1.16–2.51, *P* < 0.001) and 1.94 kPa O_2_ (95% CI = 1.32–2.65, *P* < 0.001) respectively, or ~72 hours high CO_2_ treatments, difference of means = 1.35 (95% CI = 0.61–1.92, *P* < 0.01) and 1.39 kPa O_2_ (95% CI = 0.80–2.07, *P* < 0.01) respectively (Fig. 4B). Changes in P_50_ were accompanied by changes in Hills’ number (*F* = 25.27, df = 3, *P* < 0.001). Trout acutely exposed to high CO_2_ in normoxia and hyperoxia had lower Hills’ numbers than control trout (1.93 ± 0.08, difference of means = 0.52, 95% CI = 0.30–0.74, *P* < 0.001), or ~72 hours high CO_2_ conditions (1.90 ± 0.03, difference of means = 0.49, 95% CI = 0.26–0.72, *P* < 0.001).

### Oxygen consumption rate

**Figure 5:**
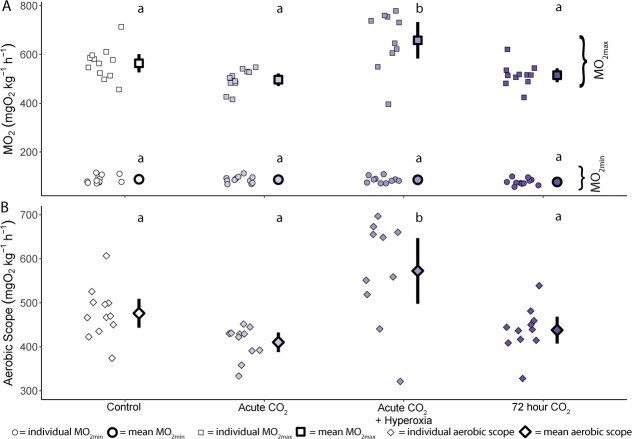
Changes in oxygen consumption of trout exposed to combined changes in environmental CO_2_ and O_2_. (**A**) Minimum O_2_ consumption (ṀO_2min_, circles) and maximum O2 consumption (ṀO_2max_, squares), (**B**) and absolute aerobic scope (diamonds) of trout exposed to control (~0.1 kPa CO_2_, ~ 21 kPa O_2_, *n* = 12), acute high CO_2_ (~30 minutes, ~ 1 kPa CO_2_, ~ 21 kPa O_2_, *n* = 11), acute high CO_2_ + hyperoxia (~30 minutes, ~ 1 kPa CO_2_, ~ 42 kPa O_2_, *n* = 10), or ~72 hour high CO_2_ (~72 hours, ~ 1 kPa CO_2_, ~ 21 kPa O_2_, *n* = 11) treatments. Each panel shows raw data points for individual trout as well as mean (bold outlined data point) and 95% confidence interval of mean (indicated by the vertical black line). Different letters above mean ṀO_2min_, ṀO_2max_ and aerobic scope of each treatment indicate significant differences in post-hoc tests (*P* < 0.05).

ṀO_2min_ of trout was not different between treatment groups (χ^2^ = 4.41, df = 3, *P* = 0.220, Fig. 5A) whereas ṀO_2max_ was (χ^2^ = 24.39, df = 3, *P* < 0.001, Fig. 5A). We observed no significant reduction in ṀO_2max_ between control trout (564.2 ± 20.4 mgO_2_ kg^−1^ h^−1^) and trout exposed to ~ 1% CO_2_ in normoxia either acutely (difference of means = 67.5 mgO_2_ kg^−1^ h^−1^, 95% CI = −15.9—150.9, *P* = 0.149) or for ~ 72 hours (difference of means = −49.1 mgO_2_ kg^−1^ h^−1^, 95% CI = −132.2—34.0, *P* = 0.399). In contrast, trout exposed to acute high CO_2_ in hyperoxia showed a ~17% increase in ṀO_2max_ when compared to control trout (difference of means = 94.21 mgO_2_ kg^−1^ h^−1^, 95% CI = 8.97–179.46, *P* = 0.025), a ~28% increase when compared to trout exposed to acute high CO_2_ in normoxia (difference of means = 161.7 mgO_2_ kg^−1^ h^−1^, 95% CI = 74.8–248.7, *P* < 0.001) and a ~24% increase compared to trout exposed to high CO_2_ for ~ 72 hours (difference of means = 143.3 mgO_2_kg^−1^ h^−1^, 95% CI = 56.0–230.5, *P* < 0.001).

**Figure 6:**
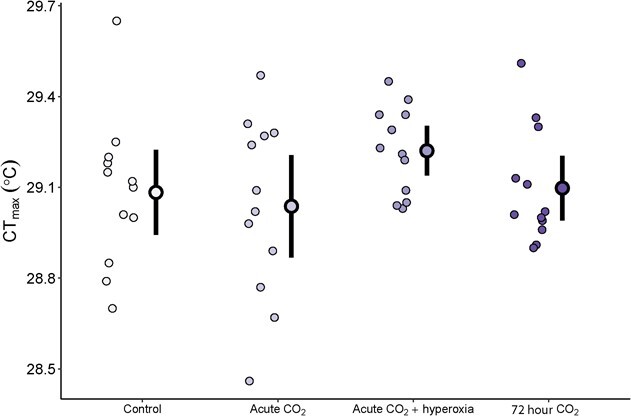
Critical thermal maximum of trout exposed to control (~0.1 kPa CO_2_, ~ 21 kPa O_2_, *n* = 12), acute high CO_2_ (~30 minutes, ~ 1 kPa CO_2_, ~ 21 kPa O_2_, *n* = 12), acute high CO_2_ + hyperoxia (~30 minutes, ~ 1 kPa CO_2_, ~ 42 kPa O_2_, *n* = 12), or ~72 hour high CO_2_ (~1 kPa CO_2_, ~ 21 kPa O_2_, *n* = 12) treatments. Individual circles represent raw data points for each treatment with the mean and 95% confidence interval of mean indicated by the bold outlined data point and vertical black line.

Changes in ṀO_2max_ between treatments were reflected in aerobic scope (χ^2^ = 24.64, df = 3, *P* < 0.001, Fig. 5B). Trout exposed to high CO_2_ in normoxia did not have lower aerobic scope than trout in control conditions (acute high CO_2_, difference of means = 60.9 mgO_2_ kg^−1^ h^−1^, 95% CI = −15.1—146.9, *P* = 0.146; ~ 72 hour high CO_2_, difference of means = 38.2 mgO_2_ kg^−1^ h^−1^, 95% CI = −42.6—119.0, *P* = 0.588). In contrast, trout exposed to acute high CO_2_ in hyperoxia showed an increase in aerobic scope of ~ 20% over trout in control conditions (difference of means = 96.3 mgO_2_ kg^−1^ h^−1^, 95% CI = 13.4–179.2, *P* = 0.017).

### Critical thermal maximum

There were no differences in CT_max_ between trout across all treatments with means ranging from 29.04 to 29.22°C across treatments (*F* = 1.42, *P* = 0.251, Fig. 6).

## Discussion

We found that acute exposure to increased CO_2_ caused a large respiratory acidosis in rainbow trout but this did not affect CT_max_. We also found that exposure of trout to elevated CO_2_ levels for 72 hours, during which trout could partially acid–base regulate, did not affect CT_max_. In combination, these results provide strong evidence that short term changes in environmental CO_2_ (exposure over hours to days) are unlikely to affect acute upper temperature tolerance in rainbow trout. Additionally, despite trout exposed to combined hyperoxia and acute high CO_2_ having increased aerobic scope this only resulted in a trend for marginally increased CT_max_, potentially contrasting with the predictions of the OCLTT hypothesis (Pörtner *et al.,* 2017; Jutfelt *et al.,* 2018).

### Impacts of CO_2_ and Hyperoxia on O_2_ supply capacity

As expected, acute exposure to high CO_2_ led to a pronounced respiratory acidosis in trout with a drop in blood pH leading to a decreased Hb–O_2_ binding affinity (i.e. an increase, almost doubling, of P_50_, Fig. 3B). We originally hypothesized that an acute acidosis would cause decreases in maximum O_2_ consumption (ṀO_2max_) precisely for this reason, i.e. reduced Hb–O_2_ binding affinity causing a reduction in the capacity for O_2_ uptake from the environment. Instead, our results show only marginal impacts of acute high CO_2_ exposure on ṀO_2max_ (~10% reduction) when compared to control trout (Fig. 5). In addition, trout from the ~ 72-hour high CO_2_ exposure group show similar ṀO_2max_ to trout from the acute high CO_2_ treatment despite complete recovery of both intracellular pH and Hb–O_2_ affinity. Combined these results suggest that changes in Hb–O_2_ binding affinity may not limit O_2_ supply capacity after exercise for trout in normoxia.

In contrast, trout exposed to acute high CO_2_ and hyperoxia exhibited higher ṀO_2max_ than any other treatment group. Several species, including rainbow trout, have previously exhibited similar increases in ṀO_2max_ when exposed to acute hyperoxia (Brijs *et al.,* 2015; McArley *et al.,* 2018, 2021), which may be a result of increased arterial pO_2_ maintaining high Hb-O_2_ saturation post-exhaustive exercise (Wood and Jackson, 1980; Milligan and Wood, 1986, 1987). McArley *et al.* (2022b) recently demonstrated this in rainbow trout where improved MO_2max_ was linked to higher levels of Hb bound O_2_ (i.e. increased Hb saturation) in hyperoxia-exposed fish. This may subsequently improve O_2_ supply to the compact myocardium and improve cardiac output (McArley *et al.,* 2021), which could also increase ṀO_2max_.

### Oxygen supply capacity and respiratory acidosis do not affect CT_max_ of trout

Despite large differences in aerobic scope between trout exposed to hyperoxia and trout exposed to normoxia, we did not observe any significant changes in CT_max_ (Fig. 6), with only a trend for CT_max_ to increase by 0.1°C in trout exposed to hyperoxia. Our results agree with several previous studies that found little or no effect of changes in aerobic scope on CT_max_ of fish (Brijs *et al.,* 2015; Ern *et al.,* 2016, 2017; Motyka *et al.,* 2017). However, our results do not match those of a recent study in which rainbow trout in hyperoxia showed a 0.87°C increase in CT_max_ (McArley *et al.,* 2022a). While some aspects of the two studies are comparable (same level of environmental O_2_ in hyperoxia treatments, similar heating rates during CT_max_ tests), there are notable differences that may explain the contrasting results, including the interactive effects of acute high CO_2_ exposure, duration of hyperoxia exposures (>22 hours vs. 0.5 hours in this study) and the acclimation temperatures of trout prior to experiments. As acute high CO_2_ exposure in normoxia did not affect CT_max_ (Fig. 6), it seems unlikely that this would prevent hyperoxia from having beneficial impacts on CT_max_. Duration of exposure to hyperoxia could play a significant role in physiological acclimation, as chronic hyperoxia exposure can result in changes in underlying metabolic systems, such as increased mitochondrial densities in tissues (Mustafa *et al.,* 2011), which improve aerobic scope when acutely re-exposed to normoxia (Skeeles *et al.,* 2022) and could facilitate increased CT_max_ when compared with fish acutely exposed to hyperoxia. Despite this, the length of hyperoxia exposures in our study compared to McArley *et al.* (2022a) is unlikely to be long enough to cause differences in physiological acclimation, as noted by Skeeles *et al.* (2022). The differing length of hyperoxia exposure does, however, seem to have caused differences in blood O_2_ transport, with McArley et al. (2021) noting a decrease in Hct in hyperoxia-exposed trout, which we did not observe in our study. Decreased Hct reduces blood viscosity, likely reducing energetic costs to the heart and thereby reducing O_2_ needed to maintain cardiac function during warming. As the heart is noted as a potential critical organ for setting the upper temperature tolerance limits of fish (Eliason *et al.,* 2011), a reduction in energetic costs to the heart (caused by reduced Hct) may contribute to the differences between our study and McArley et al. (2021).

Another possible explanation for the discrepancy between these two studies is the difference in acclimation temperature used. Trout in this study were acclimated to 15°C, whereas McArley *et al.* (2022a) acclimated trout to 10°C. Increased acclimation temperature is well known to increase the CT_max_ of species, and the mean CT_max_ recorded in our study was 2–3°C above that recorded by McArley *et al.* (2022a). In the other species in which CT_max_ has been tested during exposure to hyperoxia at two acclimation temperatures (European perch, *Perca fluviatilis*), the effects of hyperoxia were diminished at the upper temperature acclimation (Brijs *et al.,* 2015; Ekström *et al.,* 2016). Together, these results provide support for suggestions that the mechanisms responsible for determining the CT_max_ of fish may change depending on whether fish are cold- or warm-acclimated (Ern *et al.,* 2023). It is well known that in fish acclimated to increasingly higher temperatures, there is a limit above which temperature tolerance no longer increases with higher acclimation temperature (Adams *et al.,* 2022)—the so-called concrete ceiling (Sandblom *et al.,* 2016). Therefore, O_2_ supply capacity may potentially determine temperature tolerance for cold-acclimated fish, while O_2_-independent mechanisms set the ceiling of thermal tolerance for warm-acclimated fish. The reduced intraindividual variation in CT_max_ seen in trout exposed to acute high CO_2_ and hyperoxia may show evidence for a ‘concrete ceiling’ that prevented increased O_2_ supply capacity from improving CT_max_. If this is the case, it may have important ecological implications as high-temperature extremes most commonly occur in the summer, when fish are seasonally acclimated to warmer temperatures, which might result in reduced impacts of environmental hyperoxia or hypoxia on thermal tolerance limits.

As changes in aerobic scope and Hb–O_2_ binding affinity did not affect CT_max_, the critical thermal limits of rainbow trout in this study were likely determined by a mechanism other than O_2_ supply capacity. We hypothesized that acute high CO_2_ exposure may reduce the CT_max_ of trout if the mechanisms underlying the upper temperature tolerance are also affected by respiratory acidosis. Conversely, we observed no impacts of acute high CO_2_ exposures on the CT_max_ of trout (irrespective of changes in aerobic scope). This suggests that the mechanisms determining the critical temperature tolerance of trout in our study are not sensitive to pH and could provide evidence that processes relatively pH-insensitive, such as increasing fluidity of phospholipid membranes (Bowler, 2018; Chung and Schulte, 2020) leading to mitochondrial dysfunction, may be responsible for acute temperature tolerance limits. However, the upper thermal tolerance limits of fish are likely to be a result of complex interactions between multiple physiological mechanisms (Ern *et al.,* 2023), and as such, it is unlikely that a single mechanism can be identified to determine the CT_max_ of fish. Despite this, our results provide further evidence that increased environmental CO_2_ does not reduce CT_max_, and fluctuations of CO_2_ in the environment are unlikely to affect the acute temperature tolerances of rainbow trout. Whether this result can be applied generally to teleost fish is uncertain. Rainbow trout can survive across a relatively large temperature and salinity range (they are capable of moving between freshwater and seawater; Molony, 2001), which may make their physiology robust to combined changes in environmental factors. Despite this, their acid–base physiology (both in terms of capacity and regulation rate) is typical of many teleosts, and as the CO_2_ levels used in this study are likely more extreme than those fish would commonly encounter in natural environments, we believe that the lack of impacts of high CO_2_ exposure on thermal tolerance is likely to be relevant for a broad range of teleost fish.

## Conclusion

Overall, our results demonstrate that the acute upper temperature tolerance limits of rainbow trout are not affected by the respiratory acidosis caused by acute exposure to increased CO_2_. This, combined with other existing studies (Clark *et al.,* 2017; Ern *et al.,* 2017; Frommel *et al.,* 2020; Singh *et al.,* 2023), suggests that exposure to fluctuations in CO_2_ that may occur in the environment is unlikely to reduce the upper temperature tolerances of fish. We also show no effect of increased O_2_ supply capacity under high CO_2_ on CT_max_. While these results could be interpreted as disagreeing with predictions of the OCLTT (Jutfelt *et al.,* 2018), it has been argued that ultimate tolerance tests, such as CT_max_, lie beyond sustainable critical thermal limits of organisms and do not provide ideal information on whether oxygen limitation can alter sublethal thresholds, such as pejus temperatures, which provide the foundation for the OCLTT (Pörtner *et al.,* 2017). As such, experiments that combine the chronic effects of environmental O_2_ change with measurements of sublethal thresholds of thermal performance curves are needed to fully assess the utility of the OCLTT for fish. Despite this, because oxygen limitation has been shown to affect CT_max_ in some species and contrasting intraspecies results have been seen across studies, continued research is needed to fully understand what causes this variability. One avenue that should be further explored is how environmental factors affect temperature tolerance limits in fish acclimated to a range of temperatures—particularly focusing on high-temperature acclimations near the upper ceiling of a species’ CT_max_, which would be more ecologically relevant for determining the importance of interactive stressors on the thermal tolerance of fish during summer heating extremes.

## Acknowledgements

We would like to thank Paul Tyson, Rebecca Turner, Alex Bell and Richard Silcox of the Aquatic Resource Centre at the University of Exeter for their assistance with fish husbandry and the maintenance of aquarium facilities.

## Author Contributions

D.W.M. participated in the conceptualization, methodology, formal analysis, validation, investigation, writing—original draft, writing—review and editing, visualization, funding acquisition and resources. J.F. participated in the investigation and writing—review and editing. S.D.S., G.H.E., S.N.R.B. and R.W.W. participated in the supervision, funding acquisition, investigation and writing—review and editing. R.W.W. participated in the conceptualization, methodology, supervision, funding acquisition, investigation and writing—review and editing.

## Conflicts of Interest

The authors declare no conflicts of interest.

## Funding

This work was supported by a NERC GW4+ Doctoral Training Partnership studentship from the Natural Environment Research Council (NERC) (NE/L002434/1) with additional funding from CASE partner, The Centre for Environment, Fisheries and Aquaculture Science (Cefas), to D.W.M. and from the Biotechnology and Biological Sciences Research Council (BB/D005108/1 and BB/J00913X/1) and NERC (NE/H017402/1) to R.W.W.

## Data Availability

The data underlying this article are available in the University of Exeter’s data depository, ORE, at https://doi.org/10.24378/exe.5126

## Supplementary Material

Web_Material_coae026
