# Supplementary material for: Respiratory acidosis and O2 supply capacity do not affect the acute temperature tolerance of rainbow trout (Oncorhynchus mykiss)
Source: Conserv Physiol. 2024 May 16;12(1):coae026. doi: 10.1093/conphys/coae026 (PMC11109029; doi:10.1093/conphys/coae026)
Supplement: Web_Material_coae026 [file web_material_coae026.pdf]

Respiratory acidosis and O_2 supply capacity do not affect acute temperature tolerance of rainbow trout (*Oncorhynchus mykiss*)

Supplementary Material

Table S1: Measurement type, number of fish, and mean body mass of the three batches of trout used for experiments

Batch ID	Group ID	Measurement type	Number of fish	Body mass ± S.D. (g)
#1	A	Respirometry	30	205.0 ± 74.3
#2	B	Respirometry	14	189.7 ± 73.2
	C	Blood Sampling	33	368.5 ± 64.4
#3	D	CT_{max}	48	273.6 ± 68.2

Table S2: Mean ± S.D. of temperature, pH, salinity, pO_2, pCO_2, and total alkalinity (TA) in individual isolation tanks during treatments and in gill irrigation chambers at the time of blood sampling.

Treatment	Measurement	Temperature (°C)	pH (NBS)	Salinity	pO_2 (kPa)	pCO_2 (kPa)	TA (µM)
Control CO_2	Isolation chamber	14.7 ± 0.18	7.70 ± 0.07	0.12 ± 0.01	20.03 ± 0.33	0.081 ± 0.010	857 ± 65
	Gill irrigation	14.7 ± 0.31	7.61 ± 0.04	0.12 ± 0.01	19.9 ± 0.54	0.096 ± 0.004	803 ± 103
Acute high CO_2 + normoxia	Isolation chamber	15.0 ± 0.14	6.69 ± 0.02	0.12 ± 0.00	20.08 ± 0.50	0.786 ± 0.073	762 ± 58
	Gill irrigation	14.9 ± 0.12	6.61 ± 0.06	0.13 ± 0.02	18.9 ± 1.33	0.922 ± 0.172	745 ± 127
Acute high CO_2 + hyperoxia	Isolation chamber	14.9 ± 0.09	6.69 ± 0.03	0.13 ± 0.01	38.35 ± 1.15	0.834 ± 0.048	813 ± 24
	Gill irrigation	14.7 ± 0.35	6.67 ± 0.10	0.14 ± 0.01	40.20 ± 2.07	0.880 ± 0.064	834 ± 130
72 hour CO_2	Isolation chamber	15.0 ± 0.05	6.68 ± 0.04	0.12 ± 0.01	20.28 ± 0.36	0.818 ± 0.088	789 ± 68
	Gill irrigation	14.9 ± 0.14	6.66 ± 0.07	0.13 ± 0.02	19.8 ± 1.16	0.904 ± 0.220	818 ± 69

Table S3. Checklist of 53 essential criteria from Killen et al. 2021 (Killen *et al.*, 2021) for the reporting of methods for aquatic intermittent-flow respirometry

Number	Criterion and Category	Response
	**EQUIPMENT, MATERIALS, AND SETUP**	
1	Body mass of animals at time of respirometry	Mean body mass 200.1g, S.D. = 73.4 g, Minimum body mass = 91 g, Maximum body mass = 423 g
2	Volume of empty respirometers	4.515 L
3	How chamber mixing was achieved	Using a recirculating loop
4	Ratio of net respirometer volume (plus any associated tubing in mixing circuit) to animal body mass	Mean ratio = 22.56 kg/L, Min ratio = 10.67 kg/L, Max ratio = 49.6 kg/L
5	Material of tubing used in any mixing circuit	PVC with spiraled polyester yarn
6	Volume of tubing in any mixing circuit	165 mL

**7**	Confirm volume of tubing in any mixing circuit was included in calculations of oxygen uptake	Volume is included in volume of empty chamber (4.515 L)
**8**	Material of respirometer (e.g. glass, acrylic, etc.)	Acrylic
**9**	Type of oxygen probe and data recording	Pyroscience OXROB10 Robust O_2 probe
**10**	Sampling frequency of water dissolved oxygen	1 Hz
**11**	Describe placement of oxygen probe (in mixing circuit or directly in chamber)	O_2 probe was placed in mixing circuit
**12**	Flow rate during flushing and recirculation, or confirm that chamber returned to normoxia during flushing	Flow rate of recirculating loop = 170 L per hour, Flow rate of flushing = 300 L per hour
**13**	Timing of flush/closed cycles	MO_{2min} = 300 seconds flush, 240 seconds closed<br><br>MO_{2max} = 300 seconds flush, 180 seconds closed
**14**	Wait (delay) time excluded from closed measurement cycles	60 seconds
**15**	Frequency and method of probe calibration (for both 0 and 100% calibrations)	Calibrated at 100 % air saturation in beaker bubbled with air, calibrated at 0% air saturation using sodium sulphite. Calibrated before each group of fish were measured.
**16**	State whether software temperature compensation was used during recording of water oxygen concentration	Yes
	**MEASUREMENT CONDITIONS**	
**17**	Temperature during respirometry	15 °C
**18**	How temperature was controlled	Using a Grant TX150 R2 in the sump of the system (Grant Instruments, Cambridge, UK)
**19**	Photoperiod during respirometry	12L:12D
**20**	If (and how) ambient water bath was cleaned and aerated during measurement of oxygen uptake (e.g. filtration, periodic or continuous water changes)	Periodic water changes with water from recirculating system (water was sterilised by UV filter)
**21**	Total volume of ambient water bath and any associated reservoirs	~300 L
**22**	Minimum water oxygen dissolved oxygen reached during closed phases	~80 % air saturation during MO_{2min}
**23**	State whether chambers were visually shielded from external disturbance	Chambers were visually shielded from external disturbance
**24**	How many animals were measured during a given respirometry trial (i.e. how many animals were in the same water bath)	4 trout were measured during each respirometry trial (2 per water bath)
**25**	If multiple animals were measured simultaneously, state whether they were able to see each other during measurements	Fish in each water bath (2 chambers per water bath) were able to see each other during measurements
**26**	Duration of animal fasting before placement in respirometer	5 days
**27**	Duration of all trials combined (number of days to measure all animals in the study)	Respirometry trials completed in 16 days

**28**	Acclimation time to the laboratory (or time since capture for field studies) before respirometry measurements	Minimum of 1 month
	**BACKGROUND RESPIRATION**	
**29**	State whether background microbial respiration was measured and accounted for, and if so, method used (e.g. parallel measures with empty respirometry chamber, measurements before and after for all chambers while empty, both)	Background respiration was measured before and after each trial
**30**	State if background respiration was measured at beginning and/or end, state how many slopes and for what duration	Background respiration was measured for a minimum of 30 minutes
**31**	State how changes in background respiration were modelled over time (e.g. linear, exponential, parallel measures)	Changes in background respiration were modelled as linear over time
**32**	Level of background respiration (e.g. as a percentage of SMR)	Background respiration was ~2 % of MO_{2min}
**33**	Method and frequency of system cleaning (e.g. system bleached between each trial, UV lamp)	System cleaned with dilute bleach between respirometry groups
	**STANDARD OR ROUTINE METABOLIC RATE**	
**34**	Acclimation time after transfer to chamber, or alternatively, time to reach beginning of metabolic rate measurements after introduction to chamber	24 hours
**35**	Time period, within a trial, over which oxygen uptake was measured (e.g. number of hours)	16 hours
**36**	Value taken as SMR/RMR (e.g. quantile, mean of lowest 10 percent, mean of all values)	MO_{2min} calculated as mean of lowest 10 measurements due to high coefficient of variation when using q0.2 method
**37**	Total number of slopes measured and used to derive metabolic rate (e.g. how much data were used to calculate quantiles)	n/a
**38**	Whether any time periods were removed from calculations of SMR/RMR (e.g. data during acclimation, periods of high activity [e.g. daytime])	n/a
**39**	r^2 threshold for slopes used for SMR/RMR (or mean)	0.95
**40**	Proportion of data removed due to being outliers below r-squared threshold	n/a
	**MAXIMUM METABOLIC RATE**	

**41**	When MMR was measured in relation to SMR (i.e. before or after)	after
**42**	Method used (e.g. critical swimming speed respirometry, swim to exhaustion in swim tunnel, or chase to exhaustion)	Swim to exhaustion via manual chase
**43**	Value taken as MMR (e.g. the highest rate of oxygen uptake value after transfer, average of highest values)	Highest single rate of oxygen uptake value after transfer
**44**	If MMR measured post-exhaustion, length of activity challenge or chase (e.g. 2 min, until exhaustion, etc.)	Until exhaustion, defined as the cessation of burst swimming and lack of response to alight pinch of the caudal fin (usually within 3 minutes)
**45**	If MMR measured post-exhaustion, state whether further air-exposure was added after exercise	No further air exposure apart from during transfer to chamber (~15 seconds)
**46**	If MMR measured post-exhaustion, time until transfer to chamber after exhaustion or time to start of oxygen uptake recording	Time to start of O_2 measurements ~ 2 minutes
**47**	Duration of slopes used to calculate MMR (e.g. 1 min, 5 min, etc.)	2 minutes
**48**	Slope estimation method for MMR (e.g. rolling regression, sequential discrete time frames)	Entire measurement period
**49**	How absolute aerobic scope and/or factorial aerobic scope is calculated (i.e. using raw SMR and MMR, allometrically mass-adjusted SMR and MMR, or allometrically mass-adjusting aerobic scope itself)	Absolute aerobic scope calculated using allometrically scaled MO_{2min} and MO_{2max}
	**DATA HANDLING AND STATISTICS**	
**50**	Sample size	**N = 12 (control), 11 (acute high CO_2 + normoxia). 10 (acute high CO_2 + hyperoxia), 11 (chronic CO_2)**
**51**	How oxygen uptake rates were calculated (software or script, equation, units, etc.)	Using Aquaresp3
**52**	Confirm that volume (mass) of animal was subtracted from respirometer volume when calculating oxygen uptake rates	Yes
**53**	State whether analyses accounted for variation in body mass and describe any allometric mass-corrections or adjustments	Mass specific data was allometrically scaled using scaling exponents calculated as the slope of the relationship between log(MO_{2min}) or log(MO_{2max}) and log(body mass). See Figure S1.

Table S4: Mean ± S.D. of temperature, pH, salinity, pO_2, pCO_2, and total alkalinity (TA) in respirometry tanks during $\dot{M}O_{2min}$ measurements, in isolation chambers during treatments, in chase chambers during exhaustive exercise, and in respirometry tanks during $\dot{M}O_{2max}$ measurements.

Treatment	Measurement	Temperature (˚C)	pH (NBS)	Salinity	pO_2 (kPa)	pCO_2 (µatm)	TA (µM)
Control	$\dot{M}O_{2min}$	15.1 ± 0.25	7.77 ± 0.07	0.18 ± 0.05	19.53 ± 0.56	0.072 ± 0.010	854 ± 29
	Isolation chamber	14.8 ± 0.00	7.72 ± 0.03	0.13 ± 0.01	20.60 ± 0.35	0.076 ± 0.005	817 ± 26
	Chase chamber	14.9 ± 0.13	7.72 ± 0.04	0.18 ± 0.07	20.67 ± 0.17	0.077 ± 0.010	815 ± 28
	$\dot{M}O_{2max}$	15.1 ± 0.25	7.68 ± 0.03	0.19 ± 0.05	18.61 ± 0.74	0.085 ± 0.005	831 ± 17
Acute high CO_2 + normoxia	$\dot{M}O_{2min}$	15.0 ± 0.10	7.75 ± 0.05	0.17 ± 0.04	19.70 ± 0.30	0.073 ± 0.007	835 ± 31
	Isolation chamber	14.9 ± 0.12	6.72 ± 0.05	0.17 ± 0.04	20.41 ± 0.15	0.757 ± 0.128	794 ± 63
	Chase chamber	14.9 ± 0.22	6.79 ± 0.05	0.18 ± 0.04	20.66 ± 0.20	0.569 ± 0.369	813 ± 62
	$\dot{M}O_{2max}$	15.0 ± 0.10	6.73 ± 0.05	0.17 ± 0.04	18.76 ± 0.51	0.743 ± 0.114	790 ± 37
Acute high CO_2 + hyperoxia	$\dot{M}O_{2min}$	15.1 ± 0.16	7.77 ± 0.03	0.12 ± 0.01	19.72 ± 0.60	0.068 ± 0.007	806 ± 46
	Isolation chamber	14.8 ± 0.23	6.72 ± 0.05	0.12 ± 0.01	40.73 ± 3.66	0.698 ± 0.074	731 ± 36
	Chase chamber	14.9 ± 0.40	6.78 ± 0.03	0.12 ± 0.02	40.22 ± 1.00	0.592 ± 0.074	714 ± 61
	$\dot{M}O_{2max}$	15.0 ± 0.23	6.73 ± 0.06	0.12 ± 0.01	40.81 ± 1.22	0.693 ± 0.056	739 ± 49
72 hour CO_2	$\dot{M}O_{2min}$	15.1 ± 0.15	6.72 ± 0.08	0.15 ± 0.03	19.88 ± 0.33	0.776 ± 0.136	800 ± 43
	Isolation chamber	14.9 ± 0.13	6.69 ± 0.07	0.14 ± 0.03	20.42 ± 0.36	0.823 ± 0.115	796 ± 51
	Chase chamber	14.8 ± 0.20	6.78 ± 0.07	0.14 ± 0.04	19.88 ± 0.33	0.671 ± 0.140	809 ± 44
	$\dot{M}O_{2max}$	15.0 ± 0.19	6.72 ± 0.06	0.14 ± 0.04	18.76 ± 0.65	0.775 ± 0.116	803 ± 49

Table S5: Mean ± S.D. of temperature, pH, salinity, pO_2, pCO_2, and total alkalinity (TA) during treatments in isolation chambers as well as starting temperatures and heating rates of CT_{max} trials and water chemistry (using measurements at the start and end of trials) and O_2 levels (continuously measured) across CT_{max} trials.

Treatment	Measurement	Temperature (°C)	Heating rate (°C min^{-1})	pH (NBS)	Salinity	pO_2 (kPa)	pCO_2 (kPa)	TA (µM)
Control CO_2	Isolation chamber	15.2 ± 0.25	N/A	7.63 ± 0.07	0.10 ± 0.00	19.07 ± 0.30	0.089 ± 0.008	774 ± 87
	CT_{max}	15.5 ± 0.15	0.048 ± 0.001	7.61 ± 0.09	0.10 ± 0.01	18.07 ± 0.44	0.104 ± 0.024	790 ± 84
Acute high CO_2	Isolation chamber	15.2 ± 0.10	N/A	6.52 ± 0.05	0.11 ± 0.01	19.03 ± 0.48	1.096 ± 0.135	723 ± 67
+ normoxia	CT_{max}	15.7 ± 0.17	0.047 ± 0.001	6.59 ± 0.10	0.11 ± 0.01	18.24 ± 0.55	1.035 ± 0.174	740 ± 68
Acute high CO_2	Isolation chamber	15.3 ± 0.12	N/A	6.50 ± 0.10	0.10 ± 0.00	43.09 ± 2.07	1.122 ± 0.242	695 ± 59
+ hyperoxia	CT_{max}	15.7 ± 0.07	0.047 ± 0.000	6.58 ± 0.11	0.10 ± 0.00	38.60 ± 1.08	1.032 ± 0.207	715 ± 75
Chronic CO_2	Isolation chamber	15.2 ± 0.32	N/A	6.61 ± 0.07	0.11 ± 0.00	18.87 ± 0.34	0.982 ± 0.156	795 ± 37
	CT_{max}	15.7 ± 0.16	0.047 ± 0.001	6.61 ± 0.08	0.11 ± 0.00	18.35 ± 0.54	1.108 ± 0.145	833 ± 48

Table S6: Full model outputs for general linear mixed effects models (GLMM) and general linear models (GLM). Maximum likelihood ratio tests that employ chi-square statistics or F-tests were used to compare models that contained or dropped fixed effects. Estimates ± standard errors were taken from R packages used to run models. For categorical variables with multiple categories estimates ± SE are given for each category compared to the reference control category (i.e. Intercept).

Effect type	Effect name	Effect Categories	Estimate ± SE	df	X^2	P	Variance ± SD
i. Minimum oxygen consumption ($\dot{M}O_{2min}$, mgO_2kg^{-1}h^{-1})							
Conditional R^2 = 0.15, Marginal R^2 = 0.10							
**Intercept**			90.39 ± 4.49				

**Fixed**	Treatment	Acute CO_2	-4.10 ± 5.54	3	4.41	0.220	
		Acute CO_2 + Hyperoxia	-2.37 ± 5.68				
		72 hours CO_2	-12.13 ± 5.54				
**Random**	Batch						10.38 ± 3.22
ii. Maximum oxygen consumption ($\dot{M}O_{2max}$, $mgO_2kg^{-1}h^{-1}$)							
Conditional R^2 = 0.52, Marginal R^2 = 0.52							
**Intercept**			690.42 ± 25.65				
**Fixed**	Treatment	Acute CO_2	-85.33 ± 37.09	3	24.39	<0.001	
		Acute CO_2 + Hyperoxia	143.62 ± 38.04				
		72 hours CO_2	-85.15 ± 37.09				
**Random**	Batch						0 ± 0.00
iii. Absolute Aerobic Scope (AAS, $mgO_2kg^{-1}h^{-1}$)							
Conditional R^2 = 0.51, Marginal R^2 = 0.51							
**Intercept**			600.60 ± 25.21				
**Fixed**	Treatment	Acute CO_2	-81.04 ± 36.46	3	24.64	<0.001	
		Acute CO_2 + Hyperoxia	146.09 ± 37.40				
		72 hours CO_2	-73.14 ± 36.46				
**Random**	Batch						0 ± 0.00
**Effect type**	**Effect name**	**Effect Categories**	**Estimate ± SE**	**df**	**F**	**P**	
iv. CT_{max} (°C)							
R^2 = 0.09							
**Intercept**			29.08 ± 0.07				
**Fixed**	Treatment	Acute CO_2	-0.05 ± 0.09	3	1.42	0.251	
		Acute CO_2 + Hyperoxia	0.14 ± 0.09				
		72 hours CO_2	0.01 ± 0.09				

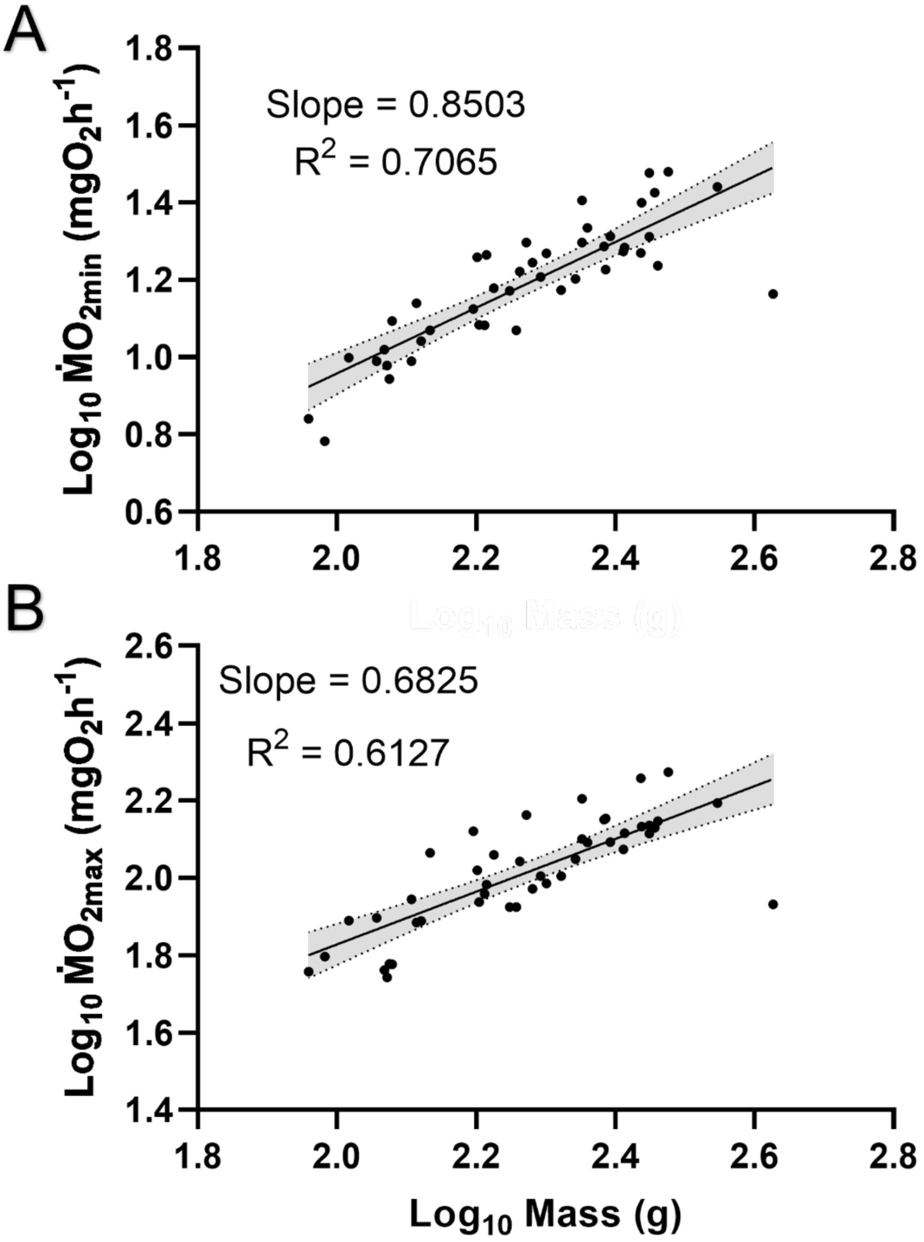

Figure S1: Linear regression between $\mathrm{Log_{10}}$(Mass) and **A.** $\mathrm{Log_{10}}(\dot{M}O_{2min})$ or **B.** $\mathrm{Log_{10}}(\dot{M}O_{2max})$ for all rainbow trout used in this study. The slope of each relationship was used as the allometric scaling exponent for mass correcting of $\dot{M}O_2$ to standard body mass of 200 g.
